# Clonal Characteristics of T-Cell Receptor Repertoires in Violent and Non-violent Patients With Schizophrenia

**DOI:** 10.3389/fpsyt.2018.00403

**Published:** 2018-08-31

**Authors:** Qiguang Li, Jiansong Zhou, Xia Cao, Qiang Liu, Qi Li, Wen Li, Xiaoping Wang

**Affiliations:** ^1^Department of Psychiatry and Mental Health Institute of the Second Xiangya Hospital, Central South University, Changsha, China; ^2^National Clinical Research Center on Mental Disorders and National Technology Institute on Mental Disorders, Hunan Key Laboratory of Psychiatry and Mental Health, Changsha, China; ^3^Department of Health Management Center, Third Xiangya Hospital, Central South University, Changsha, China; ^4^Department of Surgery, Chinese University of Hong Kong, Prince of Wales Hospital, Shatin, China; ^5^Department of Psychiatry, State Key Laboratory for Cognitive and Brain Sciences, HKU-SIRI, University of Hong Kong, Hong Kong, China

**Keywords:** schizophrenia, violence, T-cell receptor, Immune repertoire sequencing, complementarity-determining region

## Abstract

**Background:** Activated or impaired T-cell function in inflammatory and degenerative process can contribute to the risk and progression of schizophrenia. This study used immune repertoire sequencing to investigate the T-cell receptor beta variable chain (TRBV) presence in blood mononuclear cells in the violent or non-violent schizophrenic patients.

**Methods:** Ten violent and 10 non-violent schizophrenic patients and 8 matched healthy controls were enrolled. The Brief Psychiatric Rating Scale (BPRS) was used to evaluate patients' psychiatric symptoms. The level of aggression was assessed using the Modified Overt Aggression Scale (MOAS). The complementarity-determining region 3 (CDR3) of TRBV was detected using multiplex-PCR and high-throughput sequencing.

**Results:** The TCR repertoire diversity were no significant differences in the Shannon–Wiener or inverse Simpson diversity index between three groups. Principal component analysis (PCA) of TRBV composition and abundance showed that principal component 1 and principal component 2 can explain 28.88 and 13.24% of total variation, respectively. Schizophrenic patients (violent and non-violent) had significantly different V gene distribution compared to healthy controls. In particular, TRBV2 occurred at a significantly higher frequency in the violent schizophrenia group than in the non-violent schizophrenia and healthy control groups, and TRBV7-2 occurred at a significantly higher frequency in the non-violent schizophrenia group than in the violent schizophrenia and healthy control groups.

**Conclusions:** The results suggest that violent and non-violent schizophrenic patients carry abnormal T-cell receptor repertoires, and these data provide a useful clue to explore the etiology of violent behavior in schizophrenia.

## Introduction

Schizophrenia is a chronic brain disorder, the lifetime prevalence of which is nearly 1% worldwide (1). The prevalence of violent offenses in schizophrenic patients is higher than that of the general population (2–4). In China, meta-analysis from our group showed, the prevance of aggression of inpatients with schizophrenia was 35.4% (95% CI: 29.7%, 41.4%) (5). Although the etiology of violent and aggressive behavior in schizophrenia is multifactorial, genetic inheritance may strongly contribute to it (4, 6, 7). Recent studies suggested that immuno-inflammatory processes are involved in the etiology of schizophrenia (8–10) and aggressive behavior in schizophrenia (11). For example, *Toxoplasma gondii* antibodies have been observed in some schizophrenic populations (12, 13), as well as increases in maternal (14–16) and childhood infections (17). Most importantly, genetic studies also support an immune component to schizophrenia risk, the most statistically significant component is the major histocompatibility complex (MHC) region of chromosome 6 (18–21).

Activated (22, 23) or impaired (24) T-cell function in inflammatory and degenerative process has been shown to be a risk factor for schizophrenia. Moreover, biased T-cell receptor repertoires are involved in severe mental disease (25, 26). In the T-cell receptor beta variable chain (TRBV), the complementarity-determining region 3 (CDR3) is the most variable, and defines the different populations of T cells, determines the specificity by recognizing pathogen or autoantigen epitopes (27). The diversification of T cells occurs during the lymphocyte maturation process, which includes genomic rearrangements in the CDR3 variable (V), diversity (D), and joining (J) gene segments and the addition/subtraction of non-templated bases of the recombinant junctions (28).

It is estimated that the diversity of human TCR αβ pairs is nearly 2.5 × 10^18^ (29). Traditional sequencing technologies are inadequate for identification and quantification of this many T-cell antigen-receptor clonotypes. Immune repertoire sequencing, which is the large-scale sequencing of TCR repertoires, provides more distinct and detailed molecular characterization of complex sequencing targets (30). Thus, it enables the examination of the immune system at an unprecedented level (27). Using this method, we can search potential autoreactive clones and autoantigens, which may provide important information for the classification and monitoring of schizophrenia (27).

It has been proposed that immuno-inflammatory responses may enhance the risk and development of schizophrenia (14, 16, 17, 31). The immune system may influence neurometabolic, neuroendocrine, neurodevelopmental processes (32, 33), especially the effect of T-cell on cognitive function (34), learning and memory (35), social behavior (36), which are all critical etiological pathways for schizophrenia. Compared to non-violent patients, violent schizophrenic patients show disturbances in response inhibition and emotional processing (37), impaired neurocognition (38, 39), and stronger neurodevelopmental symptoms (40). Therefore, in the current study, high-throughput sequencing technology was used to analyze the characteristics and diversity of immune molecules to explore the role of T cells in the immune system-induced pathogenesis of schizophrenia. We hypothesized that schizophrenic patients would have different TCR-repertoire diversity compared to healthy controls and that violent and non-violent schizophrenic patients would have different TCR-repertoire diversity.

## Materials and methods

### Patients and sample collection

10 violent schizophrenic patients and 10 non-violent schizophrenic patients were enrolled in this study. Violent patients who had been accused of homicide were recruited from the forensic psychiatric department of a hospital in Hunan province, China. The non-violent patients, who had never displayed violent behavior, were recruited from the in- or outpatient department of the same psychiatric hospital. All patients were males who met the following criteria: 18–40 years of age, unmarried, drug-naive, no family history of psychiatric illness, and first-onset schizophrenia diagnosis according to the ICD-10. Patients with any comorbid psychiatric disorders or accompanying somatopathy were excluded. Eight healthy subjects matched for age and gender served as controls. There were no significant differences in age or years of education between three groups (Table 1).

**Table 1:** Basic demographic and clinical characteristics of violent and non-violent schizophrenia patients.

	**Violent schizophrenia (*****n*** = **10)**		**Non-violent schizophrenia (*****n*** = **10)**		**Healthy control (*****n*** = **8)**		***Statistics***		
	**M**	***SD***	**M**	***SD***	**M**	***SD***	***F***	**df**	***p***
Age	27.6	5.8	24.2	4.1	24.5	4.4	1.4	2,25	0.26
Education (years)	9.5	1.4	11.0	1.9	11.1	1.5	2,25	0.07
							***t***	**df**	**p**
BPRS									
Total score	40.7	10.5	42.1	28.5			0.1	18	0.89
Anxiety/Depression	4.4	0.7	4.5	1.6			0.2		0.86
Anergia	9.2	3.0	10.2	5.6			0.5		0.6
Thought	12.6	3.8	10.1	4.8			1.3		0.2
Disturbance	4.9	2.3	4.2	2.1			0.7		0.5
Activation	11.8	4.1	15.6	22.4			0.5		0.6
Hostility									
MOAS	25.2	3.3	0.2	0.6			23.3	18	< 0.001

*BPRS, Brief Psychiatric Rating Scale; MOAS, Modified Overt Aggression Scale*.

The study was carried out in accordance with *The Code of Ethics of the World Medical Association*. Written informed consent was obtained from all participants and their families/guardians. The study was carried out in accordance with the details of relevant guidelines and was approved by the Biomedical Ethics Board of the Second Xiangya Hospital (Approval number: 2013068).

A clinical interview was performed by a psychiatrist to confirm the diagnosis and to determine whether the patients met the inclusion and exclusion criteria. The Brief Psychiatric Rating Scale (BPRS) was used to evaluate the patients' psychiatric symptoms. The 18-item BPRS is a 7-point scale that covers a broad range of symptoms including hallucinations, delusions, and mood disturbances. Here we report the total score and the factor scores for symptoms such as anxiety/depression, anergia, thought disturbance, activation, and hostility (41).

The levels of aggression were assessed by the Modified Overt Aggression Scale (MOAS). The MOAS contains 4 differently weighted subscales, including verbal aggression, aggression against property, self-aggression, and physical aggression toward other people (42, 43). The total MOAS score was used as an overall measure of the aggression level according to the different weights of each subscale score.

### DNA extraction

A sample of peripheral blood was collected from the participants and was immediately frozen at −80°C until DNA extraction. The RelaxGene Blood DNA System (TIANGEN Biotech, Beijing, China) was used to extract DNA following the manufacturer's instructions.

### Multiplex-PCR and high-throughput sequencing of the TRBV CDR3 region

A multiplex-polymerase chain reaction (PCR) system was used to amplify rearranged CDR3 regions. The PCR amplification conditions were 96°C for 3 min; 35 cycles of 96°C denaturation for 25 s, 62°C annealing for 45 s and 72°C extension for 45 s; followed by 72°C extension for 5 min. The remaining primers in the PCR product were digested by Exo I enzyme at 37°C for 15 min, and the enzyme was then denatured at 80°C for 20 min. PCR products were purified by gel extraction and sequenced on an ABI 3730XL machine. Sequencing results were analyzed with the ATF genotyping software.

**Figure 1:**
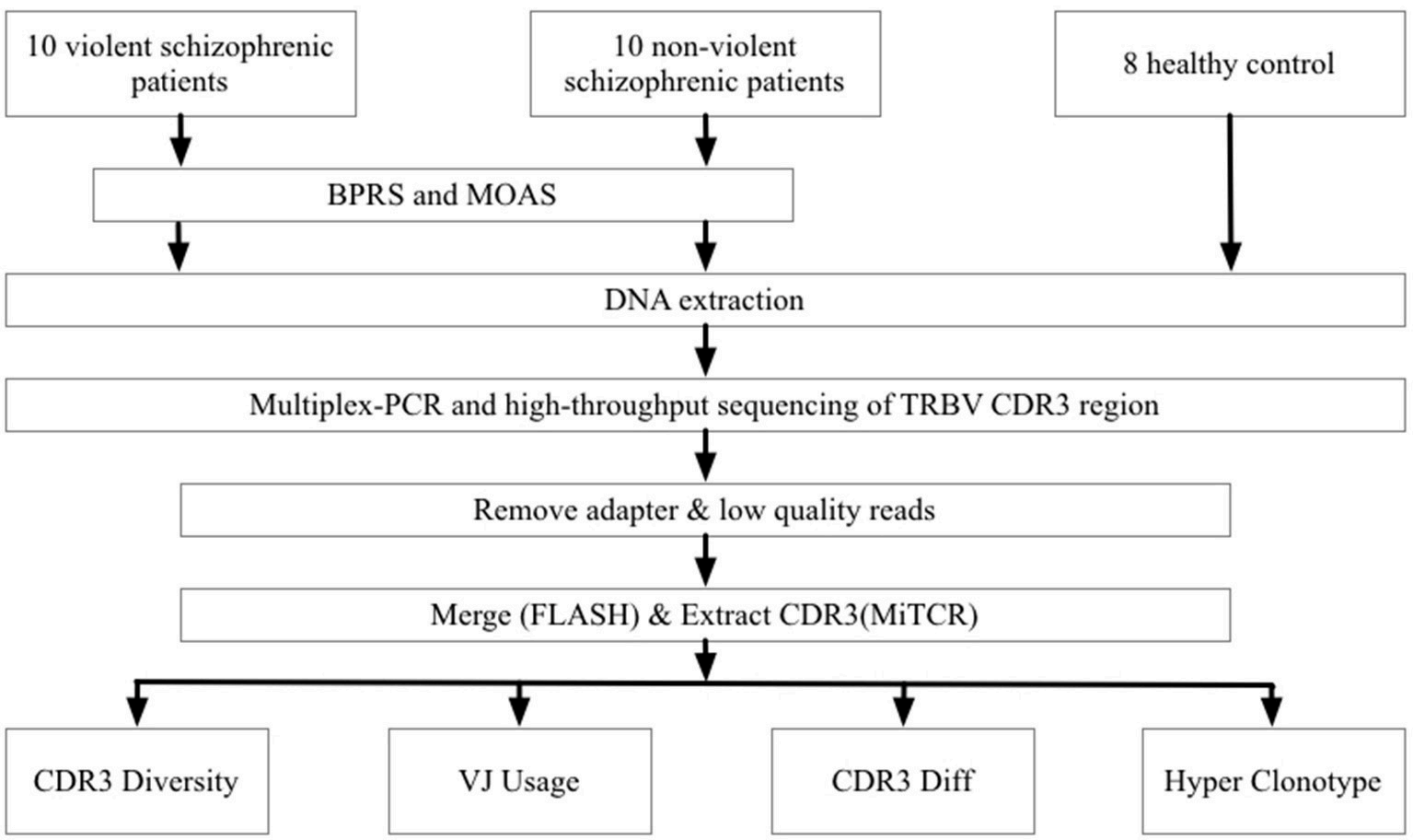
Study flowchart of experiment and data processing.

To prepare the TRBV library, 30 forward, and 13 reverse primers were designed to amplify the CDR3 region of the TRBV gene from genomic DNA template. The PCR products were purified with the AMPure XP system (Beckman Coulter, Inc, Indianapolis IN, USA) to remove PCR primers. Sequencing index sequences and adaptors were added to the library in another round of PCR with the conditions of 98°C for 1 min, 25 cycles of 98°C denaturation for 20 s, 65°C annealing for 30 s and 72°C extension for 30 s, with a final extension for 7 min at 72°C. The library was separated on an agarose gel, and the target region was isolated and purified using the QIAquick Gel Extraction kit. The PCR products were sequenced on the Illumina sequencing platform (Figure 1).

### Data analysis

Data quality was evaluated using the FastQC software. FLASH software was used to merge overlapping paired-end reads. The miTCR software, developed by MiLaboratory (http://mitcr.milaboratory.com/downloads/), was used to extract each clonotype from the CDR3 region. After sequence alignment, the expression level of each clonotype was calculated. The frequency and frequency distribution of clonotypes of the V and J gene segments, as well as the V-J gene pairs in the CDR3 region were also analyzed. To assess the TRBV repertoire diversity, the Shannon–Wiener index, the inverse Simpson diversity index, and the abundance ratios of different clonotypes were calculated (44). The V/J/VJ value of highly-expanded-clones (HECs) was calculated according to the definition of previous studies that TCR clones with a frequency of over 1% are considered to be HECs (45). Principal component analysis (PCA) was used to reduce the dimensionality of the highly expressed gene segments as described in a previous study (46). One-way ANOVA was used to compare the diversity levels among the three groups. A *p*-value of < 0.05 was considered statistically significant.

## Results

### Demographic and clinical characteristics

The mean MOAS total score of violent patients was significantly higher than that of non-violent patients (*p* < 0.001). There was no significant difference in either the total BPRS or the factor scores between the violent and non-violent patients (Table 1).

### TCR repertoire diversity among different groups

The numbers of total clonotypes and the numbers of unique clonotype in the violent patients, the non-violent patients, and the healthy control group were showed in Table 2. The number of unique clonotypes in violent schizophrenic patients was significantly higher than that in non-violent schizophrenic patients (*p* < 0.05). There were no significant differences in diversity indices such as the Shannon–Wiener and inverse Simpson diversity index between three groups (*p* > 0.05; Table 2).

**Table 2:** Diversity of T-cell receptor repertoire in the violent, non-violent and healthy control groups.

	**Violent schizophrenia**		**Non-violent schizophrenia**		**Healthy control**				
**Label**	**Mean**	**Sd**	**Mean**	**Sd**	**Mean**	**Sd**	**F**	**df**	***p***
TotalClonotype	3,016,151^***^	1,182,819	3,360,785^***^	3,721,625	12,717,786	1,198,254	44.09	2	< 0.001
UniqClonotype	76,110Δ	38,414	39,534	22,578	48,892	11,297	4.76	2	0.02
TotalHighClonotype	2,981,641^***^	1,167,216	3,341,443^***^	3,708,148	12,693,699	1,200,168	44.48	2	< 0.001
UniqHighClonotype	41,600Δ	20,956	20,192	9,356	24,805	5,221	6.35	2	0.01
Shannon's Diversity index	12.03	1.63	11.43	2.10	12.05	1.06	0.42	2	0.661
InvSimpsonDiversity	438.14	555.47	847.09	873.13	452.48	428.30	1.19	2	0.32
Normalized Shannon's Diversity index	0.75	0.09	0.76	0.14	0.77	0.07	0.16	2	0.86

*** Compare to healthy control group, p < 0.001; Δ: Compare to non-violent schizophrenia group, p < 0.01

*TotalClonotype: the total volume of clonotype expression; UniqClonotype: kinds of clonotype; TotalHighClonotype: the total expression of those clonotype express no < 2; UniqHighClonotype: kinds of clonotype express no < 2; Shannon's Diversity Index, InvSimpsonDiversity: the diversity index of clonotypes; Normalized Shannon's Diversity Index: the normalized Shannon's diversity index based on sample sequencing data*.

### TRBV CDR3 gene transcript abundance

**Figure 2:**
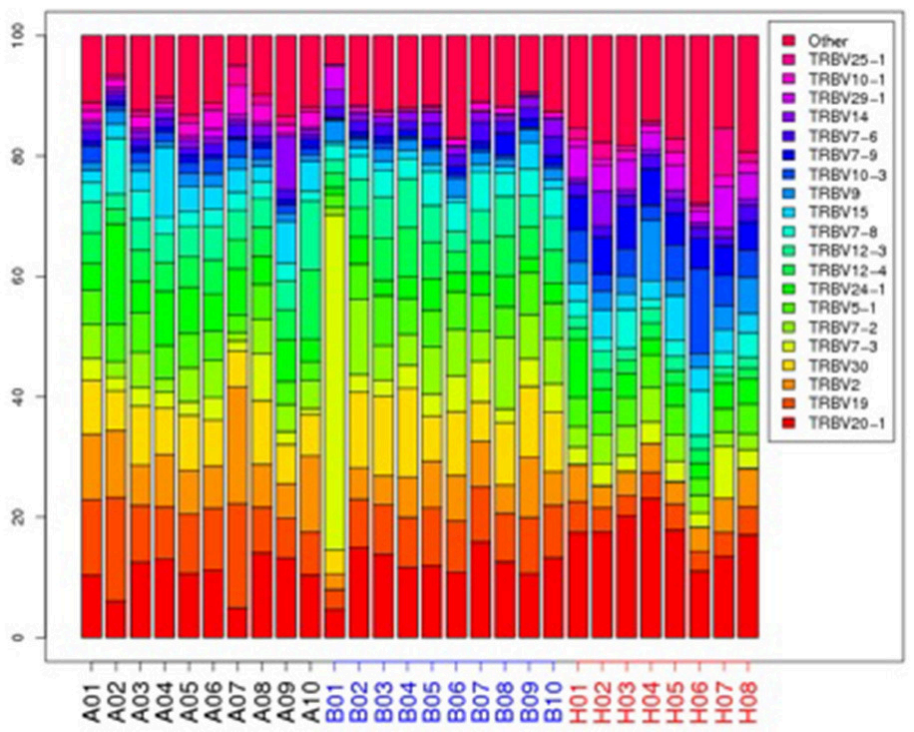
The TRBV gene usage distribution in the violent, non-violent schizophrenia and healthy control groups. A: violent schizophrenia, B: non-violent schizophrenia, H: healthy controls.

**Figure 3:**
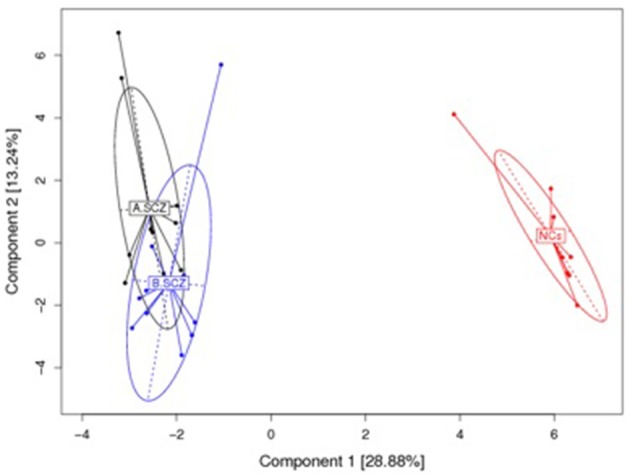
PCA results for the V gene usage plotted with respect to first and second principal components. A: violent schizophrenia, B: non-violent schizophrenia, NCs: healthy control. All V Gene Usages of the samples are plotted on to the first and second principal components (PC). The PC1 contain 28.88% information, the PC 2 contain 13.24 information. There is a significant difference between the schizophrenia patients (violent and non-violent) and healthy controls in the V gene distribution.

**Figure 4:**
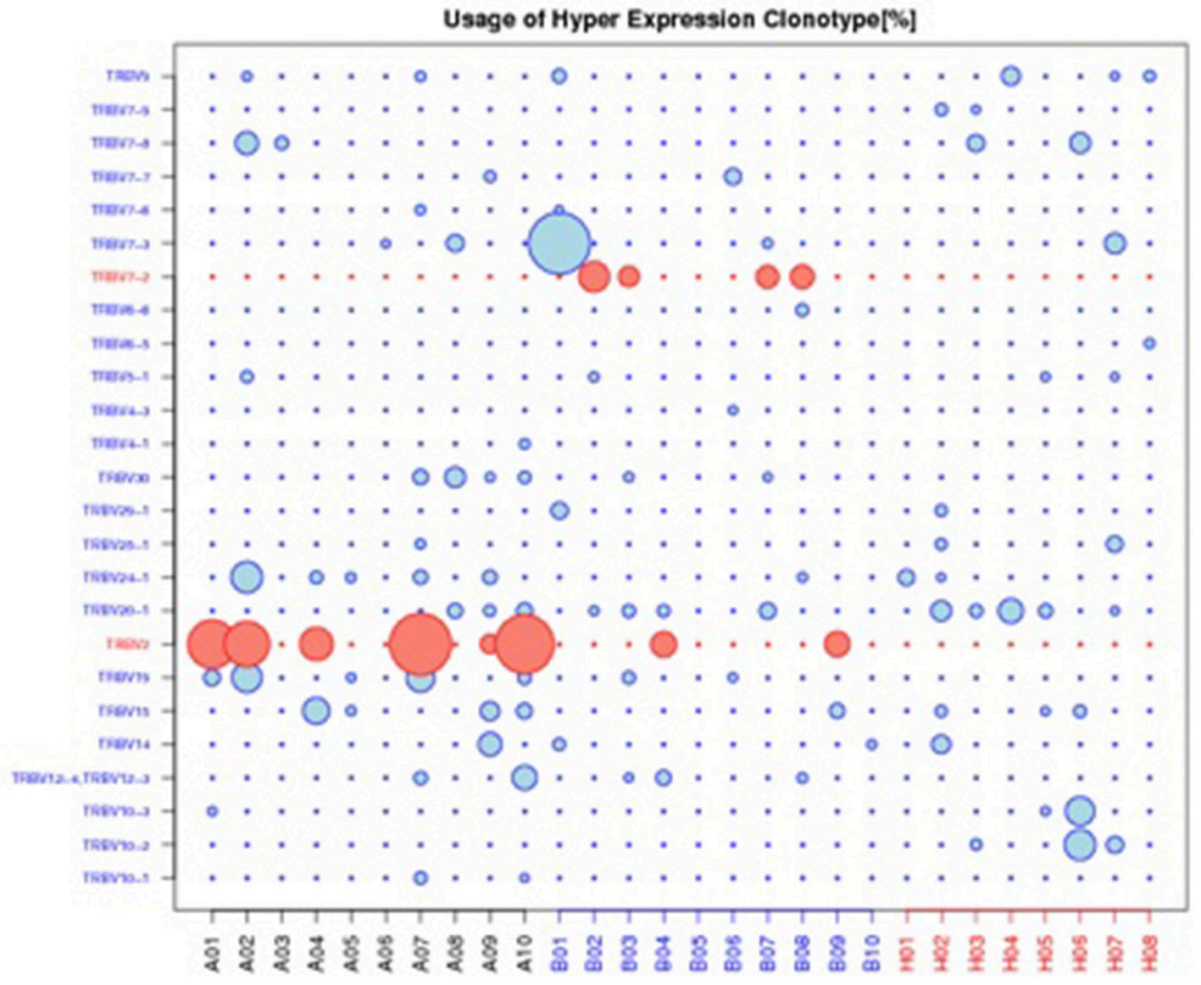
Usage of hyper expression clonotype in V gene. A: violent schizophrenia, B: non-violent schizophrenia, H: healthy control. TRBV2 was significantly higher expressed in violent schizophrenia than non-violent schizophrenia and healthy control, TRBV7-2 was significantly higher expressed in non-violent schizophrenia than violent schizophrenia and healthy control.

The top 20 most highly expressed V genes in each participant were listed in Figure 2. PCA revealed two principal components (PCs), PC1 explaining 28.88% and PC2 explaining 13.24% of the variation in the V genes (Figure 3). There were a significant differences between the schizophrenic patients (both violent and non-violent) and the healthy controls in V gene distribution (*p* < 0.01; Figure 4). In particular, TRBV2 had significantly higher levels of expression in the violent schizophrenic patients than those of both the non-violent schizophrenic patients (*p* < 0.01) and the healthy controls (*p* < 0.01), whereas TRBV7-2 had significantly higher levels of expression in the non-violent schizophrenic patients than those of both the violent schizophrenic patients (*p* < 0.01) and the healthy controls (*p* < 0.01). Similar patterns were found in the J and V-J genes in all participants, and there were no significant differences in J and V-J gene pair distribution among the three groups.

## Discussion

To our knowledge, this is the first study using immune repertoire sequencing to investigate the characteristics and polymorphisms of the TRBV in violent and non-violent schizophrenic patients. The study found that schizophrenic patients (violent and non-violent) have different V gene distributions compared to those of healthy controls. In particular, the number of unique clonotypes in the violent schizophrenic patients was significantly higher than that in the non-violent schizophrenic patients. TRBV2 showed significantly higher expression in the violent schizophrenic patients, and TRBV7-2 showed significantly higher expression in the non-violent schizophrenic patients.

Each T-cell bears a unique T-cell receptor in order to recognize a specific antigen-derived peptide. Recognizing MHC-bound peptides through TCRs, T cells mount the immune response in the adaptive immunity process. Some alterations in T-cell function in schizophrenia have been demonstrated, including reduced proliferative responses to stimulation, significant changes in transcripts associated with the cell cycle, intracellular signaling, oxidative stress and metabolis (47), and activation of T-cell networks (48). In addition, it has also been reported that altered immune function caused by T-cell molecular changes is associated with first-onset schizophrenia (49).

The finding that V gene distribution in schizophrenic patients is different from healthy controls provides evidence that the dysregulation of T cells is associated with schizophrenia. TRBV provides the diversity and composition of the entire set of antigen receptors on T cells, which has an extraordinary impact on health and disease. Normally, without any antigen stimulation, T cells are in a positive polyclonal state, and TRBV is randomly rearranged. However, in the case of disease, specific antigen stimulation causes targeted TRBV subfamily rearrangement and dramatic clonal expansion. Therefore, the dominant T-cell clone may suppress the cloning of other T cells, resulting in a decrease in immune function (27). Clarifying TCR repertoire characteristics may help to explore the role of the immune system in the pathogenesis of schizophrenia and aid in the diagnosis and personalized treatment of patients.

Recently, Li et al. reported immune factors (C3 and Th17-related cytokines) were related to schizophrenia and aggressive behavior (11), it suggested some immune factors would be potential biomarkers for aggressive behavior with schizophrenia. The immune repertoire sequencing is a new tool for deciphering mechanisms of autoimmunity. As the variety of TRBV genes have been indentified (50), and different distribution of TRBV subfamilies may result in decrease or increase some immune function (51). To our knowledge, there are some studies reported a significant skewed TRBV repertoire occurred, such as in major depressive disorder (26), and the systemic lupus erythematosus (27), acute graft-versus-host disease (52), and the maternal–fetal interface (53). In this study, the violent schizophrenic patients had higher numbers of unique expressed clonotypes than non-violent patients, and violent patients expressed higher levels of TRBV2, whereas non-violent patients expressed higher levels of TRBV7-2. TRBV subfamilies exhibit pedigree polymorphism and abnormal clonal proliferation under stimulation with specific antigens may cause a specific immune response. The highly oligoclonal nature of T cells in these patients may be associated with the different reactivity of each patient. The results of this study showed that violent schizophrenic patients have a different distribution of TRBV subfamily abnormalities, suggesting the abnormal expression of some TRBV subfamilies in PBMCs may be associated with the immune pathogenesis of schizophrenia. This provides a valuable clue to explore potential violence-specific T-cell antigens involved in immune-related schizophrenia pathogenesis, which may provide insight for the development of novel diagnostics and targeted immunotherapy. Till now, however, few studies reported the relation between immune factors and TRBV. Further study is needed to identify the function of the TRBV subfamilies evaluated *in vivo* and *in vitro*.

## Limitations

This study is limited in some respects. First, as a heterogeneous disorder, schizophrenia produces an extraordinary variety and wide array of symptoms. Although, we used the BPRS to control the symptoms, violent and non-violent patients with schizophrenia may not be from uniform subtypes. Second, the sample size was small, and increasing the sample in future studies would provide opportunities for identifying additional oligoclonal or even monoclonal hyperplasia subfamilies. Finally, the function of this process and the clonal proliferation of T-cells in the body is largely unclear; further research is needed to conclusively reveal the molecular mechanisms of T-cell signaling.

## Author contributions

JZ and XW designed the study, QgL and WL wrote the protocol. JZ, XC managed the literature searches and analyses. QgL wrote the first draft of the manuscript. All authors contributed to and have approved the final manuscript.

### Conflict of interest statement

The authors declare that the research was conducted in the absence of any commercial or financial relationships that could be construed as a potential conflict of interest.
